# Structure-Based Virtual Screening for KHK-A Inhibitors with Anti-Hepatocellular Carcinoma Activity

**DOI:** 10.3390/ph18121865

**Published:** 2025-12-06

**Authors:** Jiang-Yi Zhu, Xiao-Yang Han, Zi-Ying Zhou, Yue-Yue Guo, Hao-Tian Duan, Jia-Jia Shen, Si-Tu Xue

**Affiliations:** 1 Institute of Medicinal Biotechnology, Chinese Academy of Medical Sciences & Peking Union Medical College, Beijing 100050, China; 2 West China Hospital of Stomatology, Sichuan University, Chengdu 610065, China

**Keywords:** KHK-A, virtual screening, structure-based drug design

## Abstract

**Background:** Hepatocellular carcinoma (HCC) is the sixth most common malignant tumor worldwide and is associated with a poor prognosis. Oxidative stress is a key factor in the occurrence and progression of HCC. KHK-A, a key protein in the oxidative stress pathway, plays an important role in various cancers. This study aimed to discover small-molecule inhibitors targeting KHK-A through structure-based virtual screening, evaluate their therapeutic effects on HCC, and explore the potential of KHK-A as a therapeutic target for HCC. **Methods:** Based on the crystal structure of KHK-A, potential small-molecule inhibitors (HK1 to HK-24) were screened from the SPECS database using the Discovery Studio (DS) 2019 software. The effects of these compounds were evaluated through molecular docking and cellular experiments. **Results:** The screened compound HK-4 significantly inhibited HCC cell proliferation, migration, and invasion ex vivo. The half-maximal inhibitory concentrations (IC_50_) of HK-4 in HepG2, PLC/PRF/5, and HuH7 cells were 22.54 µM, 23.91 µM, and 23.38 µM, respectively. HK-4 induced G1 phase arrest and apoptosis, and reduced the protein levels of p-AKT and p-mTOR in the PI3K-AKT signaling pathway. **Conclusions:** Through structure-based virtual screening, this study identified HK-4, a small-molecule inhibitor of KHK-A with anti-HCC activity. Its mechanism of action is closely related to the regulation of the PI3K-AKT signaling pathway. This finding provides experimental evidence supporting KHK-A as a therapeutic target for HCC and offers a new direction for the development of novel anti-HCC drugs.

## 1. Introduction

Liver cancer ranks as the sixth most common malignant tumor globally, with approximately 870,000 new cases reported in 2022, and is the third leading cause of cancer-related deaths, accounting for about 760,000 fatalities [1,2,3,4]. Surgical resection is the primary treatment for liver cancer; however, only 15% of patients are eligible for tumor resection, and the five-year recurrence rate remains as high as 60–70% [5]. The advent of molecular targeted therapies has improved liver cancer treatment, but the prognosis for advanced-stage patients remains poor due to the inevitable development of drug resistance during therapy [6].

Since the approval of sorafenib for advanced liver cancer in 2007, the field of multi-target tyrosine kinase inhibitors (TKIs) has expanded. Lenvatinib (approved in 2018) has emerged as a new first-line treatment option, while regorafenib (2017), cabozantinib (2019), and ramucirumab (2019) have contributed to prolonged survival in second-line settings. Meanwhile, although immunotherapy for HCC has shown promise, its efficacy still requires improvement [7]. In monotherapy, lenvatinib as a first-line treatment demonstrates efficacy comparable to sorafenib, with a median overall survival (mOS) of 13.6 months, slightly improved over sorafenib’s 12.3 months [8]. Additionally, the combination therapy of atezolizumab plus bevacizumab (approved in 2020) achieves an mOS of 19.2 months [9]. The heterogeneity of HCC is a key factor contributing to resistance against both TKIs and immunotherapy, as well as poor prognosis [10,11]. Liver cancer exhibits significant genetic diversity, and this heterogeneity makes it difficult for single-agent therapies to target all cancer cell subpopulations, particularly therapy-resistant tumor-initiating cells with stem-like properties [12]. Although strategies combining immune checkpoint inhibitors (ICIs) with TKIs show potential, complex immune evasion mechanisms driven by tumor heterogeneity continue to limit treatment efficacy [13]. Therefore, both cellular heterogeneity and microenvironment complexity collectively contribute to the limitations of current therapies, underscoring the urgent need to further investigate the mechanisms underlying HCC development and progression, and to expand treatment options to improve patient survival and prognosis.

Oxidative stress is a pathological condition characterized by elevated intracellular reactive oxygen species, leading to dysregulation of redox signaling and oxidative damage to macromolecules [14]. As a key hallmark of cancer, oxidative stress significantly promotes the initiation and progression of HCC through multiple mechanisms, including increased cellular damage and genetic mutations, influence on specific gene variations, and activation of related signaling pathways. It also participates in regulating tumor cell growth, remodeling the microenvironment, inducing immunosuppression, and promoting angiogenesis [15,16]. Ketohexokinase (KHK) is the first rate-limiting enzyme in fructose metabolism. KHK-A, one of the isoforms produced by alternative splicing of the KHK gene, is predominantly expressed in various cancer tissues and exhibits minimal fructose phosphorylation activity [17]. Studies indicate that KHK-A plays important roles in multiple cancers: in colorectal cancer, it is associated with fructose metabolism and promotes cancer cell proliferation and metastasis; in gastric cancer, under high glucose conditions, it is activated by fructose produced via the polyol pathway, promoting epithelial-mesenchymal transition (EMT) and enhancing cell migration and invasion; in HCC, it phosphorylates p62 to activate the Nrf2 signaling pathway, aiding cancer cells in adapting to metabolic stress [17,18,19,20].

Studies have shown that elevated KHK-A phosphorylation levels are associated with reduced survival rates in patients, highlighting the significance of KHK-A overexpression in the severity of HCC [21]. Considering the link between the oxidative stress protein KHK-A and the progression of HCC malignancy, and recognizing the scarcity of treatment targets, this study seeks to evaluate the impact of KHK-A inhibitors on liver cancer treatment. It specifically looks at their potential to intervene in tumor metastasis and recurrence. Additionally, the study examines the feasibility of targeting KHK-A as a therapeutic approach for HCC and preliminarily evaluates KHK-A’s suitability as a drug target.

## 2. Results

### 2.1. Identification of the KHK-A Active Site for Virtual Screening Based on Structural Analysis

**Figure 1:**
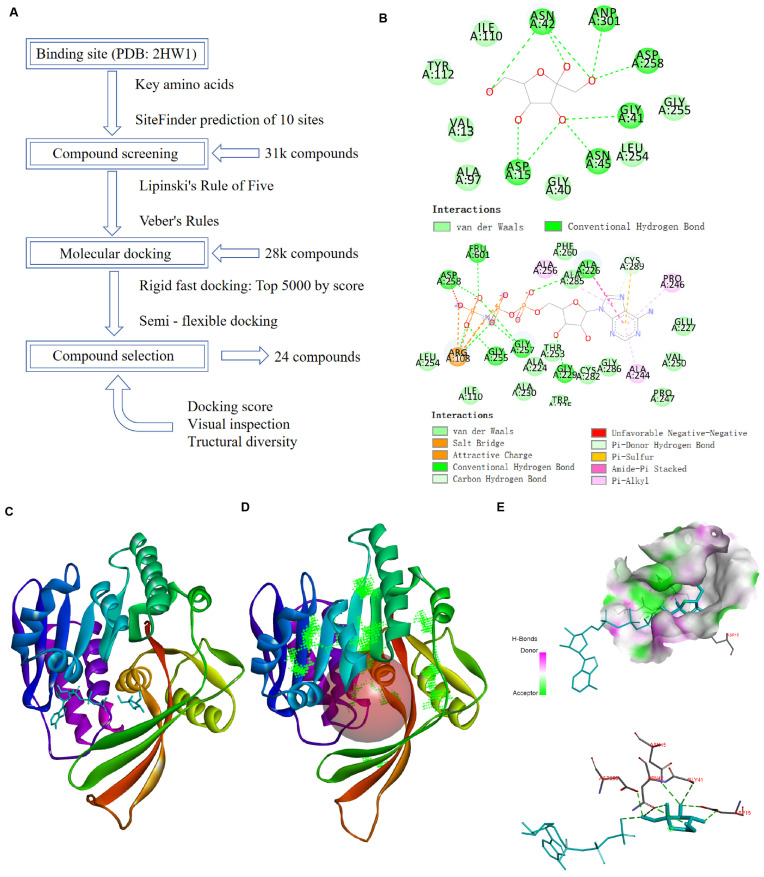
Structure-based virtual screening of KHK-A inhibitors: (**A**) Workflow of the small-molecule screening process. (**B**) Two-dimensional interaction diagram of KHK-A with the co-crystallized small molecule. (**C**) Schematic representation of the crystal structure of KHK-A in complex with the co-crystallized small molecule. (**D**) Diagram illustrating the active site of KHK-A, identified based on key amino acids, alongside the 10 potential sites predicted by DS 2019 software. (**E**) Close-up view of the KHK-A active site, identified via key amino acids, in complex with the co-crystallized small molecule. Key amino acid residues interacting with the small molecule are labeled. The small molecule is depicted in blue.

A compound library comprising 30,936 compounds from the SPECS database was selected for high-throughput screening to identify potential small molecules targeting KHK-A. Compounds that violated Lipinski’s rule of five or Veber’s rules were excluded from the library. The filtered compounds subsequently underwent virtual docking (Figure S1). The screening workflow is illustrated in Figure 1A. Structure-based virtual screening for novel KHK-A inhibitors requires the identification of the cavity where small molecules bind to KHK-A. The crystal structure of the KHK-A ternary complex (PDB: 2HW1) reveals interactions between the substrate fructose, an ATP analog, and key amino acid residues of the KHK-A protein (Figure 1B,C). The binding pocket for fructose is situated at the extremity of the enzyme’s active site crevice. The five hydroxyl groups of fructose establish direct hydrogen bonds with specific amino acid residues within the protein (Asp15, Gly41, Asn42, Asn45, and Asp258), utilizing both the main chain and side-chain atoms [22]. Furthermore, Tyr112 is suspected to assist in the entry of fructose into the binding site. At the other end of the crevice, the base and ribose portions of AMP-PNP are bound. The hydrophobic region of the base is nestled closely between the main chains of Ala226 and Glu227, as well as the side chain of Cys289. The ribose hydroxyl groups engage in weak hydrogen bonding with the Sγ atom of Cys282 and the oxygen atom of Gly229. Mutations such as Gly40 to Arg have been found to inactivate the KHK-A isoform and result in significant insolubility when produced in E. coli, while the mutation of Ala43 to Thr decreases the protein’s stability relative to the wild type; these mutations affect residues that are integral to the fructose-binding region [23]. Asp258 functions as a base by removing a proton from the O1 hydroxyl group of fructose, enabling O1 to carry out a nucleophilic attack on the γ-phosphate of ATP, yielding fructose-1-phosphate, which is crucial for protein phosphorylation processes [24]. Therefore, Gly40, Ala43, Tyr112, Asp258, and Cys282 were identified as key residues defining binding site 1. Subsequently, the potential binding regions of the KHK-A protein were calculated using the Define Site module in the Discovery Studio (DS) 2019 software. As shown in Table 1, 10 potential binding sites (sites 2–11) were predicted for KHK-A. In this study, the optimal docking active site was determined by comparing the CDOCKER_INTERACTION_ENERGY of fructose and ATP analogs at different sites, thereby providing a basis for subsequent experimental validation. CDOCKER_INTERACTION_ENERGY represents the non-bonded interaction energy between the receptor and ligand, where a more negative value indicates stronger ligand–receptor binding and greater stability of the ligand within the binding pocket. When fructose and ATP analogs were docked into these regions, it was found that the substrates could hardly access sites 4, 5, and 7. In contrast, site 1, which was identified based on previous literature, exhibited the most favorable binding energy and was therefore selected as the active site for subsequent docking studies (Figure 1D). When the substrates were docked back into site 1, interactions with surrounding amino acids were observed (Figure 1E), further validating the reliability of the key residues reported in the literature.

### 2.2. Structure-Based Discovery of KHK-A Inhibitors Assisted by Virtual Screening

**Figure 2:**
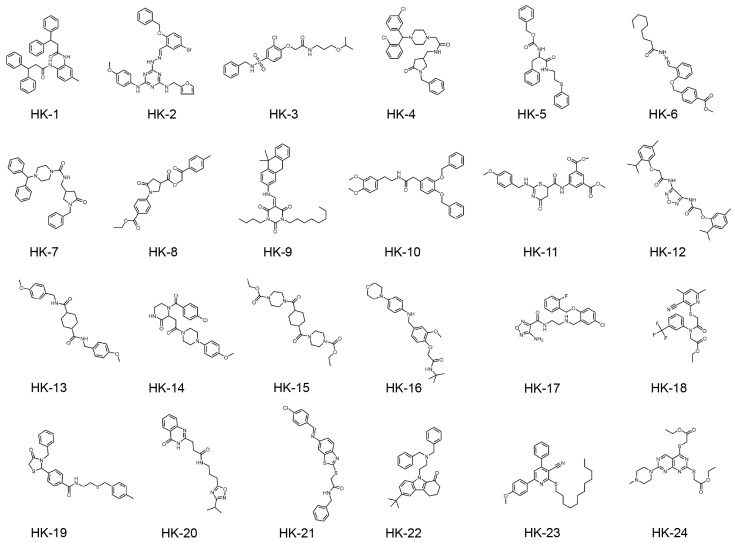
Chemical structures of HK-1 to HK-24.

**Figure 3:**
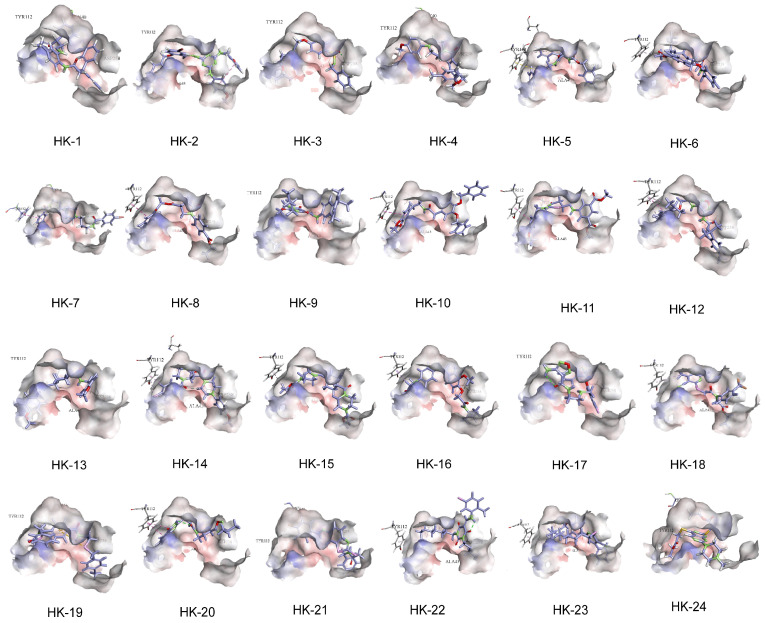
Schematic diagram of the binding of selected compounds to the KHK-A protein.

In conducting molecular docking studies, the X-ray crystal structure of KHK-A, with a resolution of 2.10 Å (PDB code: 2HW1), was obtained from the Protein Data Bank. The structure underwent preparation following conventional methods. This involved the elimination of co-crystallized water molecules and the resolution of structural anomalies within the protein, including gaps in loops, alternative conformations, and missing atoms. These adjustments were carried out using the “Clean Protein” and “Prepare Protein” features available in Discovery Studio. Hydrogen atoms were added, and protonation states were generated at pH 7.0. Based on previous studies, the binding pocket was defined near residues Gly40, Ala43, and Asp258, encompassing a spherical region with a radius of 9 Å. Molecular docking was conducted with the LibDock module within Discovery Studio 2.5, a tool that enables the swift and rigid docking of numerous ligands to a specific target protein. The compounds were sorted based on their LibDock scores, and the top 5000 were further optimized using the CHARMm force field (Table S1). Virtual screening was conducted using CDOCKER to dock all prepared ligands into the defined active site, and the optimal docking conformation of each compound was visualized. Protein–ligand interactions were analyzed using Discovery Studio 2.5. Based on a comprehensive evaluation of binding energy data, interactions with key residues in the binding pocket, and structural diversity, 24 compounds were selected for ex vivo biological evaluation (Table 2, Tables S3 and S4). In the virtual screening workflow, commercial compound libraries often consist of a series of derivatives, where the same scaffold may be enumerated multiple times. Including all of them in experimental testing would occupy limited ex vivo evaluation slots while providing information on only a single scaffold. Therefore, during the screening process, we prioritized compounds with significant structural diversity, encompassing multiple structural classes such as phthalimide, triazine, and diphenylmethylpiperazine (Table S2, Figure 2). When these compounds were docked back into the active site, most exhibited interactions with the selected key amino acid residues (Figure 3).

### 2.3. Evaluation of the Efficacy of Structure-Based KHK-A Inhibitors

**Figure 4:**
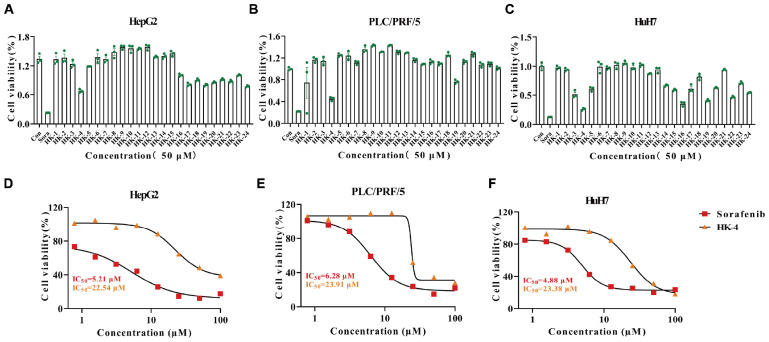
Discovery of HK-4, a KHK-A Active Site Inhibitor: (**A**–**C**) Cell viability of HepG2, PLC/PRF/5, and HuH7 cell lines following treatment with KHK-A inhibitors at a single concentration (50 µM) for 48 h. (**D**–**F**) Effects of HK-4 at various concentrations on the proliferation of HepG2, PLC/PRF/5, and HuH7 cells after 48 h of treatment.

Initially, the cell viability of HepG2, PLC/PRF/5, and HuH7 cells was determined for these 24 compounds at a concentration of 50 µM (Figure 4A–C). Among them, compound HK-4 exhibited the most potent inhibitory effect. Subsequently, the half-maximal inhibitory concentration (IC_50_) of HK-4 and the positive control drug, sorafenib, was measured using the Cell Counting Kit-8 (CCK-8) assay. The results demonstrated that the IC_50_ values for HK-4 and sorafenib were 22.54 µM and 5.21 µM, respectively, in HepG2 cells (Figure 4D); 23.91 µM and 6.28 µM, respectively, in PLC/PRF/5 cells (Figure 4E); and 23.38 µM and 4.88 µM, respectively, in HuH7 cells (Figure 4F).

### 2.4. Detection of the Effects of HK-4 on the PI3K-AKT Pathway, HCC Cell Cycle, and Apoptosis

**Figure 5:**
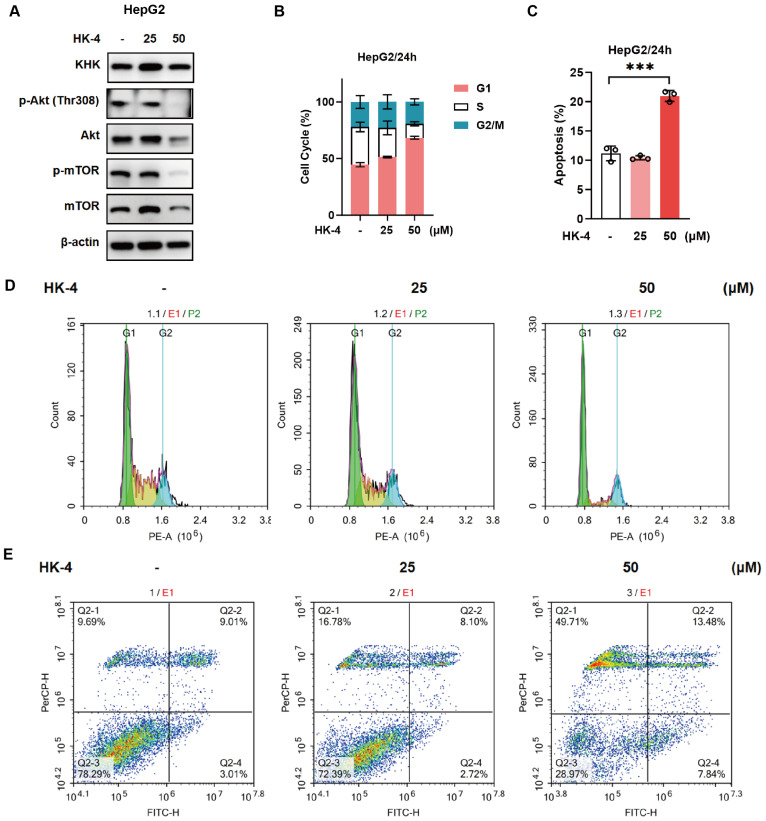
Analysis of the effects of HK-4 on the HCC cell cycle, apoptosis, and related pathways: (**A**) Effect of HK-4 on the protein expression of the PI3K-AKT signaling pathway. (**B**,**D**) Effect of HK-4 on the HepG2 cell cycle. (**C**,**E**) Effect of HK-4 on HepG2 cell apoptosis. *** *p* < 0.001.

Given that KHK-A possesses biological functions promoting tumor cell growth, proliferation, and metastasis, and considering that the aforementioned experiments have confirmed that the KHK-A active site inhibitor HK-4 exhibits significant inhibitory effects on hepatoma cell proliferation, flow cytometry was subsequently employed to detect changes in the cell cycle distribution of HepG2 cells before and after HK-4 treatment. The results demonstrated that as the concentration of HK-4 increased, the proportion of HepG2 cells in the G1 phase significantly increased, while the proportions of cells in the S and G2/M phases decreased. This confirmed that treatment with HK-4 for 24 h could dose-dependently induce G1 phase arrest in HepG2 cells (Figure 5B,D). Similarly, in experiments detecting apoptosis by flow cytometry, it was found that 50 μM HK-4 significantly increased the proportion of HepG2 cells in early and late apoptosis, indicating that a high concentration of HK-4 exhibits considerable activity in inducing tumor cell apoptosis (Figure 5C,E). Subsequently, Western blot analysis was performed to further examine changes in protein levels in HepG2 cells following HK-4 treatment. The results indicated that after 24 h of HK-4 treatment, the protein levels of p-AKT and p-mTOR were significantly reduced. This suggests that HK-4, by inhibiting the KHK-A protein, further reduces the activation state of the PI3K-AKT signaling pathway within tumor cells, thereby exerting its antitumor efficacy (Figure 5A). In addition, during the 200-nanosecond molecular dynamics simulation, the HK-4 and protein complex exhibited good structural stability. The trends in Rg and RMSD changes indicated that the complex maintained a certain degree of structural integrity throughout the simulation (Figure S2).

### 2.5. HK-4 Inhibits Migration and Invasion of Hepatocellular Carcinoma Cells

**Figure 6:**
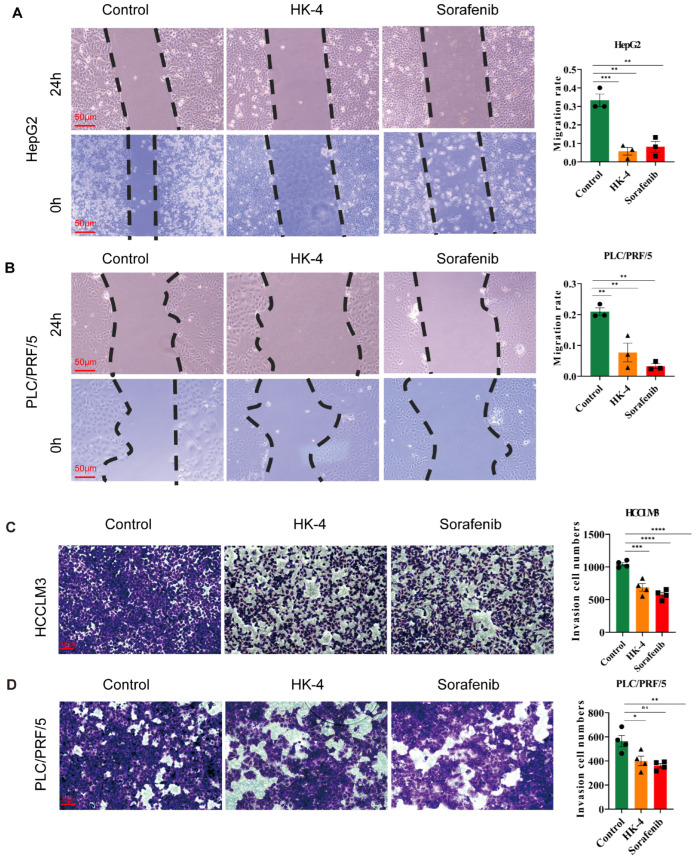
HK-4 suppresses the invasion and migration of hepatocellular carcinoma cells: (**A**,**B**) Representative images and quantitative results of the migration assay for HepG2 and PLC/PRF/5 cells treated with HK-4 (10 µM) or sorafenib (5 µM). Scale bar: 50 µm. Comparisons among multiple groups were analyzed by one-way ANOVA. (**C**,**D**) Representative images and quantitative results of the invasion assay for HCCLM3 and PLC/PRF/5 cells treated with HK-4 (10 µM) or sorafenib (5 µM). Scale bar: 20 µm. * *p* < 0.05, ** *p* < 0.01, *** *p* < 0.001, **** *p* < 0.0001.

Given that HK-4 has been identified to suppress the PI3K-AKT signaling pathway, we proceeded to explore how derivatives of HK impact the migration and invasion of HCC cells. We initiated our investigation with a scratch wound assay. The findings indicated that the application of HK-4 at a concentration of 10 µM or sorafenib at 5 µM notably decreased the migration capacity of HepG2 and PLC/PRF/5 cells (Figure 6A,B). In addition, a Transwell assay was conducted to evaluate the anti-HCC invasion effect of HK-4. The results demonstrated that HK-4 at 10 µM significantly decreased the number of invading cells, indicating that even at a low concentration, HK-4 could impair the invasive ability of HCCLM3 and PLC/PRF/5 cells (Figure 6C,D). Taken together, these findings indicate that HK-4 also exhibits potent anti-HCC activity by suppressing the migration and invasion of HCC cells.

## 3. Discussion

This study focuses on the discovery of small-molecule inhibitors of KHK-A and their therapeutic effects on HCC. KHK-A, a key protein in the oxidative stress pathway, plays a central role in the initiation and progression of HCC. Previous studies have primarily explored the function of KHK-A through methods such as gene knockout or overexpression [17]. In contrast, based on the crystal structure of KHK-A, this study identified its active site and screened the inhibitor HK-4 from a compound library. Experimental results demonstrated that HK-4 significantly inhibits the proliferation, migration, and invasion abilities of HCC cells, and this inhibitory effect is closely associated with the functional regulation of the PI3K-AKT signaling pathway. Moreover, the results of molecular dynamics studies not only help to verify the reliability of the docking results but also effectively evaluate the binding stability of the complex and the kinetic characteristics of the interactions. This study is the first to regulate oxidative stress-related signaling pathways from a chemical biology perspective by utilizing the binding of a small-molecule compound to the KHK-A protein. This finding not only provides strong experimental evidence supporting KHK-A as a potential therapeutic target but also preliminarily reveals the feasibility of modulating KHK-A with small molecules to intervene in tumor progression.

Through virtual screening and ex vivo experimental validation, we discovered that HK-4 not only exhibits significant inhibitory effects on hepatoma cells but also contains a diphenylmethylpiperazine scaffold in its chemical structure, which is notably distinct from known antitumor drug scaffolds. We found that HK-4 has a difference of at least 10% from all the antitumor compounds reported on the SCI Finder platform, indicating that HK-4 represents a novel antitumor scaffold. In previous antitumor drug development, many studies have focused on known drug targets and validated scaffolds. Novel scaffolds with antitumor activity may possess unique mechanisms of action and hold promise for overcoming the limitations of existing drugs [25,26]. The structural novelty of HK-4 is expected to contribute to addressing current challenges in HCC treatment, such as drug resistance and poor prognosis. However, HK-4 currently exhibits only moderate activity, remaining significantly distant from the criteria for a lead compound or clinical candidate. To overcome this limitation, the following follow-up studies are planned: Design and synthesis of 20–30 derivatives to significantly enhance pharmacological efficacy through systematic structure–activity relationship optimization; Evaluation of the synergistic/sensitizing effects of HK-4 series derivatives with first-line TKIs or immune checkpoint blockers, providing data to support clinical positioning strategies for either monotherapy or combination therapy.

The low oxygen, or hypoxic, conditions within the tumor microenvironment are a significant feature of HCC. These conditions can trigger drug resistance in cancer cells by activating hypoxia-inducible factors (HIFs). By employing agents that target HIFs, it’s possible to diminish the tumor cells’ adaptive reactions to low-oxygen environments, which in turn can boost the effectiveness of TKIs [10]. Studies have shown that EF24 and sorafenib exhibit synergistic effects in an orthotopic HuH-7 xenograft model, significantly enhancing the efficacy of sorafenib [27]. Furthermore, oxidative stress drives the high expression of immune checkpoint molecules through the aryl hydrocarbon receptor (AhR) signaling network, establishing a critical mechanism for tumor immune evasion and providing potential strategies for targeted intervention, such as the combination of antioxidants with ICIs [28]. Although existing TKIs and ICIs have demonstrated certain efficacy in HCC treatment, drug resistance remains a major challenge. This study found that the KHK-A inhibitor HK-4 can modulate oxidative stress-related pathways, suggesting that HK-4 has the potential to be used in combination with existing TKIs and ICIs to form multi-target treatment plans. Such combination strategies may exert synergistic effects through multiple mechanisms, thereby improving therapeutic outcomes and reducing the incidence of drug resistance.

In summary, this study not only reveals the potential of KHK-A as a therapeutic target but also identifies an antitumor small molecule, HK-4, with a novel scaffold. This discovery provides new guidance for the development of anti-hepatoma drugs and aids future exploration of more antitumor agents with entirely new scaffolds.

## 4. Materials and Methods

### 4.1. Construction of a Small-Molecule Database

High-throughput screening was conducted to identify potential small molecules targeting KHK-A. The SPECS database (http://www.specs.net), containing approximately 350,000 compounds, and a library of 31,000 small molecules downloaded from the ZINC database, were used for experimental screening. The three-dimensional coordinates of all compounds were imported into the molecular module available in DS 2019 for further structural filtering and virtual screening. The SPECS database was filtered to exclude compounds that did not comply with Lipinski’s and Veber’s rules, resulting in the removal of approximately 2400 small molecules. Ultimately, 28,000 small molecules remained for subsequent molecular docking studies.

### 4.2. Protein Preparation and Molecular Docking

For the docking analysis, the X-ray crystal structure of KHK-A (PDB ID: 2HW1) was utilized. The protein underwent standard preparation steps, which included the removal of co-crystallized water molecules and the resolution of structural issues like incomplete loop regions using Discovery Studio’s “Prepare Protein” feature. This process entailed the removal of alternative conformations, the addition of hydrogen atoms, and the assignment of protonation states at pH 7.4. Following this, potential binding sites within the protein were identified using the Define Site module. The molecular docking was executed with the Libdock module in Discovery Studio version 2.5, where all compounds were scored and ranked according to their Libdock scores. The top 5000 highest-scoring molecules were chosen for further minimization using the CHARMm force field. The virtual screening involved docking all prepared ligands into the identified active site using CDOCKER to determine the most favorable docking conformation of the compounds.

### 4.3. Molecular Dynamics

We used DS 2019 to extract the receptor and ligand data and complete the missing atoms of the protein, and then used Gromacs to perform molecular dynamics simulations and result analysis of the protein and ligand under the CHARMM force field. The topology file and initial coordinate file were obtained for the protein receptor, selecting the 8 CHARMM all-atom force field. During the simulation process, the steps of adding a box and solvent, adding ions and neutralizing the charge, energy minimization, NVT and NPT equilibration, and molecular dynamics simulation were carried out in sequence. In the result analysis stage, the changes in RMSD of the protein + ligand, the changes in RMSF of the protein, and the changes in the radius of gyration of the protein were calculated.

### 4.4. Cell Lines and Culture Conditions

The HepG2, HCCLM3, HuH7, and PLC/PRF/5 cell lines were obtained from the China Center for Type Culture Collection (CCTCC). All cell lines were authenticated by STR analysis (Promega, Madison, WI, USA) and confirmed to be free of mycoplasma contamination. All hepatocellular carcinoma (HCC) cells were cultured in DMEM medium (Invitrogen, Carlsbad, CA, USA) supplemented with 10% fetal bovine serum (FBS, Invitrogen, Carlsbad, CA, USA) and 1% penicillin–streptomycin. All cells were maintained in a humidified incubator at 37 °C with 5% CO_2_. Culture media and additives were purchased from Gibco (Grand Island, NY, USA).

### 4.5. CCK-8 Assay

To perform the Cell Counting Kit-8 (CCK-8) assay, cells were plated into 96-well plates at a concentration of 3000 cells per well. Following a 24 h incubation period, the cells were exposed to sorafenib at a concentration of 20 µM and a KHK-A inhibitor at 50 µM for a duration of 48 h. Subsequently, cell viability was determined using the CCK-8 kit (TargetMol, C0005) (Boston, MA, USA). To assess the impact of HK-4 and HK-22 on the growth of HepG2, PLC/PRF/5, and HuH7 cells, these cells were treated with varying concentrations of HK-4 and HK-22 (ranging from 0.78 to 100 µM) for 48 h. Subsequently, 10 µL of the CCK-8 reagent was introduced into each well. After a 2 h incubation at 37 °C, the absorbance was measured at 450 nm using a spectrophotometric plate reader. The IC_50_ values were calculated through nonlinear regression analysis with GraphPad Prism 9.0 software.

### 4.6. Detection of Apoptosis Rate by Flow Cytometry

To evaluate the apoptotic effect of compound HK-4 on tumor cells, an Annexin V/PI dual-staining method was employed. HepG2 cells were plated at a density of 5 × 10^5^ cells per well in 6-well plates and incubated with HK-4 at concentrations of 0, 25, or 50 µM for 24 h at 37 °C. Post-treatment, cells were detached using trypsin, rinsed with PBS, and then suspended in 100 µL of binding buffer. Afterward, 5 µL of Annexin V was added to the cells, which were then kept in the dark for 30 min. Subsequently, 5 µL of PI was introduced, and the cells were further incubated in the dark for an additional 30 min. The cells were then mixed with 400 µL of binding buffer, passed through a filter, and the apoptotic cells were quantified using flow cytometry analysis.

### 4.7. Cell Cycle Analysis by Flow Cytometry

To determine the impact of HK-4 on tumor cell cycle arrest, we conducted a PI staining assay. HepG2 cells were plated at 5 × 10^5^ cells per well in 6-well plates and treated with different doses of HK-4 (0, 20, or 50 µM) for 24 h at 37 °C. Post-treatment, cells were harvested with trypsin, fixed in 70% ethanol, and stored at −20 °C. The fixed cells were then washed in PBS and suspended in 400 µL of PBS. After adding 4 µL of RNase and incubating in a 37 °C water bath for 30 min to digest RNA, 4 µL of PI staining solution was added. The cells were stained in the dark for 60 min. Flow cytometry was used to analyze the cell cycle distribution among the treated cells.

### 4.8. Western Blotting Analysis

HepG2 cells were plated at a density of 5 × 10^5^ cells per well in 6-well plates and incubated with various doses of the HK-4 compound for 24 h at 37 °C. Post-incubation, the cells were harvested and subjected to lysis using 100 µL of RIPA buffer. Protein concentration was determined with a BCA protein assay kit. For Western blot analysis, samples were denatured by heating at 100 °C in a metal bath for 15 min. Subsequently, the process of gel electrophoresis, membrane transfer, and detection was carried out.

### 4.9. Wound Healing Assay

We employed the scratch wound assay to evaluate the migration capacity of cells. The cells were plated in 6-well plates and allowed to grow for 24 h. Subsequently, a clear wound was introduced into the cell monolayer in each well with a 200 µL pipette tip, followed by triple rinsing with PBS to remove debris. The cells were incubated in FBS-free DMEM and exposed to HK-4 (10 µM), HK-22 (10 µM), or sorafenib (5 µM). Post 24 h treatment, images of the wounded areas were captured with an inverted microscope from Olympus, Tokyo, Japan. The closure of the wound was measured by analyzing the distance between the cell fronts using ImageJ v1.53t (National Institutes of Health, Bethesda, MD, USA).

### 4.10. Invasion Assay

For evaluating cellular invasion, we utilized 24-well Transwell plates featuring 8.0 µm polycarbonate filters (Millipore, SCWP04700, Burlington, MA, USA), which were coated with 60 µL of Matrigel (BD Matrigel Matrix, 354230,San Jose, CA, USA, with 40 µL applied per well). A total of 2 × 10^5^ HCCLM3 or PLC/PRF/5 cells were suspended in serum-free medium containing either HK-4 (10 µM), HK-22 (10 µM), or sorafenib (5 µM), and this cell suspension was added to the individual inserts. The inserts were placed into the wells containing DMEM medium supplemented with 10% FBS. After a 24 h period, non-invading cells on the upper side of the inserts were removed, and the inserts were treated with PBS, fixed with 4% formaldehyde for 20 min, and stained with 0.1% crystal violet for an additional 20 min. Finally, photographs of the wells were taken, and the stained cells were counted in four randomly chosen fields of view to assess invasion.

### 4.11. Statistical Analysis

For statistical evaluation, GraphPad Prism version 9 was utilized. The results are depicted either as average values ± standard error of the mean (SEM) and are derived from a minimum of three separate trials. To compare two groups, an unpaired, two-tailed Student’s *t*-test was applied. For evaluating differences across multiple groups, a one-way analysis of variance (ANOVA) was conducted. As a rule, each experiment included at least three biological replicates. The significance levels were categorized as follows: * *p* < 0.05, ** *p* < 0.01, *** *p* < 0.001; “ns” denotes a non-significant difference.

## Figures and Tables

**Table 1:** Predicted binding sites of substrates in the KHK-A complex and the corresponding -CDOCKER energies (kcal/mol).

Site	XYZ	-CDOCKER_ INTERACTION_ ENERGY (kcal/mol)	Site	XYZ	-CDOCKER_ INTERACTION_ ENERGY (kcal/mol)
1	29.60, 17.24, −50.83	99.622	7	39.37, 28.84, −51.33	-
2	32.87, 16.84, −63.83	74.1467	8	21.87, 16.84, −58.08	65.5311
3	26.87, 26.87, −45.83	81.4457	9	32.37, 27.84, −65.33	89.9186
4	37.37, 28.09, −41.58	-	10	20.18, 4.35, −50.47	57.6797
5	28.76, 25.82, −52.85	-	11	19.12, 18.59, −52.83	57.6797
6	37.78, 36.52, −53.66	75.1432			

**Table 2:** The CDOCKER binding energy data of compounds HK-1 to HK-24 (kcal/mol).

Name	-CDOCKER_ ENERGY (kcal/mol)	-CDOCKER_ INTERACTION_ ENERGY (kcal/mol)	Name	-CDOCKER_ ENERGY (kcal/mol)	-CDOCKER_ INTERACTION_ ENERGY (kcal/mol)
HK-1	55.5566	68.9031	HK-13	42.5118	66.3195
HK-2	55.9431	61.3881	HK-14	48.9848	65.0435
HK-3	56.8529	60.4107	HK-15	46.5891	64.9229
HK-4	52.3159	63.5466	HK-16	47.3542	60.4784
HK-5	52.2516	62.7731	HK-17	47.5509	54.0555
HK-6	52.4523	73.0539	HK-18	53.5868	59.7725
HK-7	51.0916	63.111	HK-19	35.7782	51.3377
HK-8	40.5939	50.0084	HK-20	45.5042	53.1368
HK-9	32.0048	57.5651	HK-21	24.6067	58.425
HK-10	57.7976	74.0963	HK-22	41.2059	52.1573
HK-11	52.8556	59.9795	HK-23	22.1092	47.6071
HK-12	50.0547	61.626	HK-24	36.8692	59.2522

## Data Availability

Data will be made available upon request.

## Supplementary Materials

The following supporting information can be downloaded at: https://www.mdpi.com/article/10.3390/ph18121865/s1, Figure S1: Workflow overview of molecular docking and ligand optimization; Figure S2: Molecular-dynamics analysis of the protein–HK-4 complex; Table S1: Summary of small-molecule docking results from the LibDock virtual-screening stage; Table S2: Chemical names and CAS registry numbers for hit compounds HK-1 to HK-24; Table S3: Interaction modes of HK-1–HK-24 with amino-acid residues in the protein active site; Table S4: Hydrogen-bond distances between HK-1–HK-24 and amino-acid residues in the protein active site.
