# Supplementary material for: Structure-Based Virtual Screening for KHK-A Inhibitors with Anti-Hepatocellular Carcinoma Activity
Source: Pharmaceuticals (Basel). 2025 Dec 6;18(12):1865. doi: 10.3390/ph18121865 (PMC12736281; doi:10.3390/ph18121865)
Supplement: Supplementary file 1 [file pharmaceuticals-18-01865-s001.zip › pharmaceuticals-3941169-supplementary.pdf]

Structure-Based Virtual Screening for KHK-A Inhibitors with Anti-Hepatocellular Carcinoma Activity

Jiang-Yi Zhu 1,†, Xiao-Yang Han 2,†, Zi-Ying Zhou 1, Yue-Yue Guo 1, Hao-Tian Duan 1, Jia-Jia Shen 1,* and Si-Tu Xue 1,*

1. Institute of Medicinal Biotechnology, Chinese Academy of Medical Sciences & Peking Union Medical College, Beijing 100050, China
2. West China Hospital of Stomatology, Sichuan University; Chengdu 610065, China

* Correspondence: xuesitu@imb.pumc.edu.cn (S.-T.X.); shenjiajia@imb.pumc.edu.cn (J.-J.S.)

† These authors contributed equally to this work.

Supplementary Materials

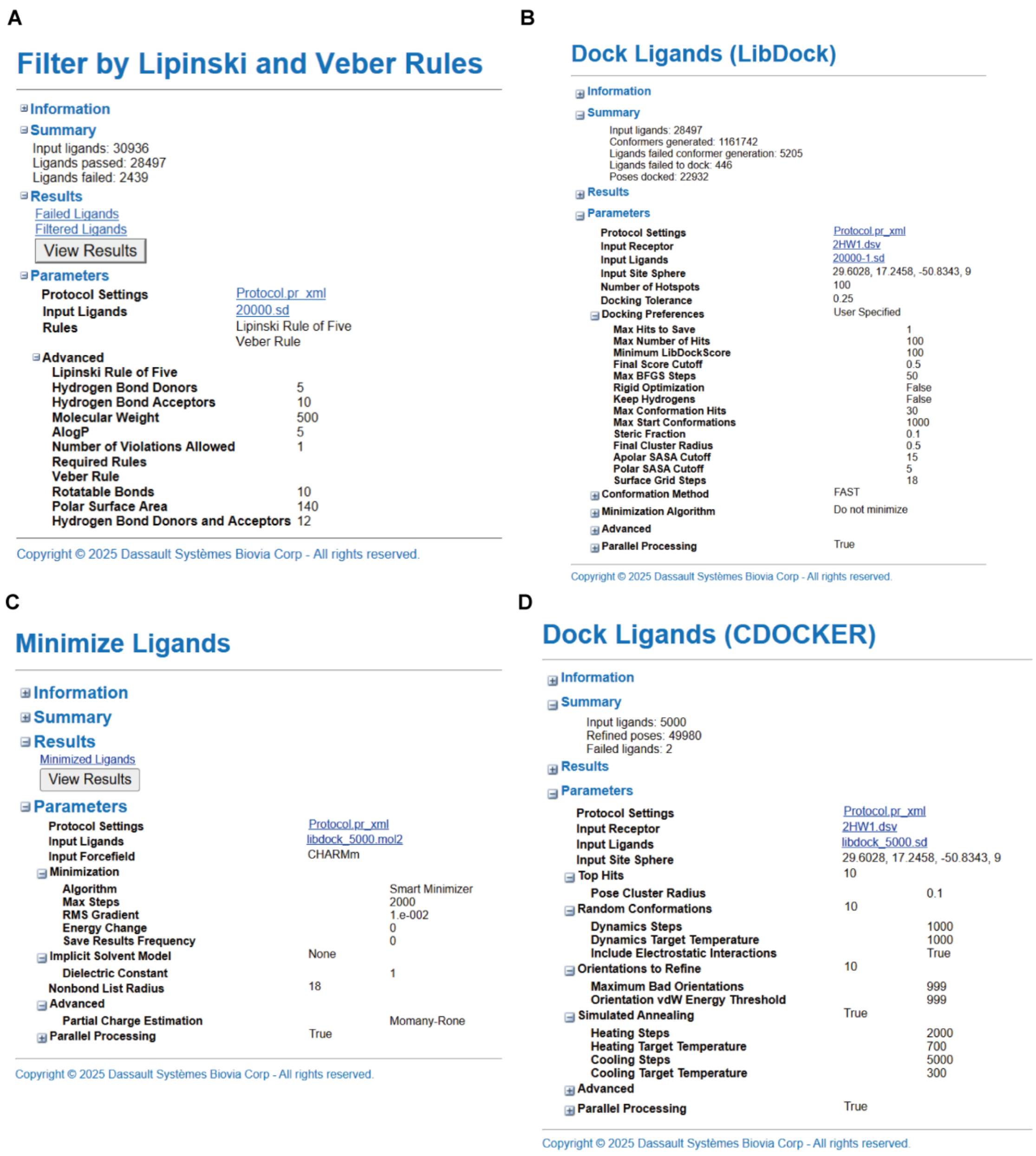

Figure S1. Overview of Molecular Docking and Ligand Optimization Process. (A) Filtering by Lipinski and Veber Rules. (B) Docking of Ligands with LibDock. (C) Docking of Ligands with CDOCKER. (D) Ligand Minimization Process.

Table S1. Results of Small Molecule Docking at the LibDock Virtual Screening Stage.

Index	LibDockScore	Molecular_PolarSurfaceArea	RelativeEnergy	AbsoluteEnergy
1	167.994	73.72	19.9051	60.7483
2	167.994	69.56	19.9051	60.7483
3	165.304	78.85	8.40773	95.2117
4	165.304	82.09	8.40773	95.2117
5	165.304	78.85	8.40773	95.2117
6	165.304	82.09	8.40773	95.2117

7	165.304	82.09	8.40773	95.2117
8	162.955	73.72	16.3484	58.4016
9	162.955	73.72	16.3484	58.4016
10	162.955	69.56	16.3484	58.4016
11	162.886	69.56	8.16277	49.7318
12	162.886	73.72	8.16277	49.7318
13	162.886	73.72	8.16277	49.7318
14	162.591	152.49	6.47885	50.062
15	162.316	77.11	13.5512	94.3503
16	162.23	53.74	12.9242	129.457
17	160.322	78.38	8.19644	83.886
18	159.811	76.41	2.11541	105.694
19	158.948	107.88	0.102858	91.8835
20	158.849	111.78	6.32022	70.3613
……	……	……	……	……
4,981	116.724	82.28	0	65.923
4,982	116.724	82.28	0	65.923
4,983	116.724	113.81	13.3878	114.298
4,984	116.724	99.59	2.97456	101.513
4,985	116.722	67.43	2.26567	50.1626
4,986	116.72	67.42	13.9438	104.968
4,987	116.717	70.93	18.5823	107.709
4,988	116.717	92.35	2.40206	83.8696
4,989	116.717	92.35	2.40206	83.8696
4,990	116.716	78.79	0.603651	54.392
4,991	116.709	84.76	1.72723	112.392
4,992	116.709	84.76	1.72723	112.392
4,993	116.701	44.7	0	65.8348
4,994	116.689	95.32	19.4996	118.456
4,995	116.689	99.11	8.19755	57.0013
4,996	116.688	58.59	17.3547	54.3007
4,997	116.686	64.22	13.0085	45.8295
4,998	116.685	54.55	9.82101	45.3276
4,999	116.685	63.21	5.74831	67.6208
5,000	116.683	90.95	0	92.7645

Table S2. Summary of Chemical Names for HK-1 to HK-24.

Number	CAS Number	Compound Name
HK-1	445253-55-4	N-{2-[(3,3-diphenylpropanoyl)amino]-4-methylphenyl}-3,3-diphenylpropanamide
HK-2	311783-07-0	2-(benzyloxy)-5-bromobenzaldehyde [4-[(2-furylmethyl)amino]-6-(4-methoxyanilino)-1,3,5-triazin-2-yl]hydrazone

HK-3	723244-33-5	2-{4-[(benzylamino)sulfonyl]-2-chlorophenoxy}-N-(3-isopropoxypropyl)acetamide
HK-4	156640-14-1	N-[(1-Benzyl-5-oxo-3-pyrrolidinyl)methyl]-4-[(2-chlorophenyl)(3-chlorophenyl)methyl]-1-piperazineacetamide
HK-5	1009053-18-2	benzyl 1-benzyl-2-oxo-2-{[2-(phenylsulfanyl)ethyl]amino}ethylcarbamate
HK-6	581779-68-2	methyl 4-{[2-(2-nonanoylcarbohydrazonoyl)phenoxy]methyl}benzoate
HK-7	156640-12-9	4-benzhydryl-N-[(1-benzyl-5-oxo-3-pyrrolidinyl)methyl]-1-piperazinecarboxamide
HK-8	774187-88-1	2-(4-methylphenyl)-2-oxoethyl 1-[4-(ethoxycarbonyl)phenyl]-5-oxo-3-pyrrolidinecarboxylate
HK-9	353282-41-4	1-butyl-5-{[(10,10-dimethyl-9,10-dihydro-2-anthracenyl)amino]methylene}-3-octyl-2,4,6(1H,3H,5H)-pyrimidinetrione
HK-10	2745-33-7	2-[3,4-bis(benzyloxy)phenyl]-N-[2-(3,4-dimethoxyphenyl)ethyl]acetamide
HK-11	577695-75-1	dimethyl 5-[({2-[(4-methoxybenzyl)amino]-4-oxo-5,6-dihydro-4H-1,3-thiazin-6-yl}carbonyl)amino]isophthalate
HK-12	512796-72-4	N,N'-1,2,5-oxadiazole-3,4-diylbis[2-(2-isopropyl-5-methylphenoxy)acetamide]
HK-13	941438-10-4	1-N,4-N-bis[(4-methoxyphenyl)methyl]cyclohexane-1,4-dicarboxamide
HK-14	1025057-76-4	4-(4-chlorobenzoyl)-3-{2-[4-(4-methoxyphenyl)-1-piperazinyl]-2-oxoethyl}-2-piperazinone
HK-15	817566-92-0	diethyl 4,4'-[1,4-cyclohexanediyldi(carbonyl)]di(1-piperazinecarboxylate)
HK-16	666209-22-9	N-(tert-butyl)-2-(2-methoxy-4-{[4-(4-morpholinyl)anilino]methyl}phenoxy)acetamide
HK-17	880807-36-3	4-amino-N-[2-({5-chloro-2-[(2-fluorobenzyl)oxy]benzyl}amino)ethyl]-1,2,5-oxadiazole-3-carboxamide
HK-18	667884-59-5	ethyl [{[(3-cyano-4,6-dimethyl-2-pyridinyl)sulfanyl]acetyl}-3-(trifluoromethyl)anilino]acetate
HK-19	361199-27-1	N-(2-{[(4-methylphenyl)methyl]sulfanyl}ethyl)-4-[4-oxo-3-(phenylmethyl)-1,3-thiazolidin-2-yl]benzamide
HK-20	1809472-03-4	2-Quinazolinepropanamide, 3,4-dihydro-N-[3-[3-(1-methylethyl)-1,2,4-oxadiazol-5-yl]propyl]-4-oxo- (ACI)
HK-21	311774-58-0	N-benzyl-2-({6-[(4-chlorobenzylidene)amino]-1,3-benzothiazol-2-yl}sulfanyl)acetamide
HK-22	300804-66-4	6-tert-butyl-9-[2-(dibenzylamino)ethyl]-2,3,4,9-tetrahydro-1H-carbazol-1-one
HK-23	333341-06-3	2-(dodecylsulfanyl)-6-(4-methoxyphenyl)-4-phenylnicotinonitrile
HK-24	667884-08-4	ethyl {[2-[(2-ethoxy-2-oxoethyl)sulfanyl]-7-(4-methyl-1-piperazinyl)pyrimido[4,5-d]pyrimidin-4-yl]sulfanyl}acetate

Table S3. Interaction Modes of HK-1 to HK-24 with Amino Acid Residues at the Protein Active Site.

HK-1

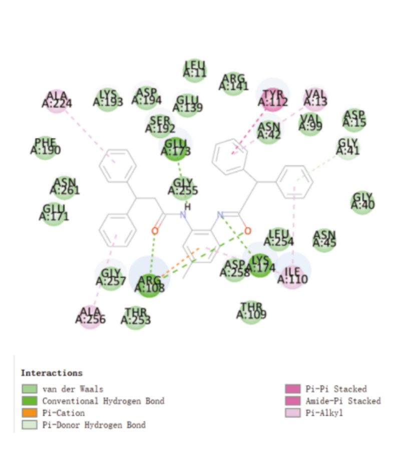

HK-2

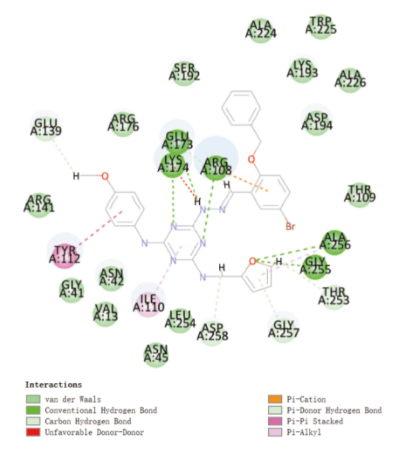

HK-3

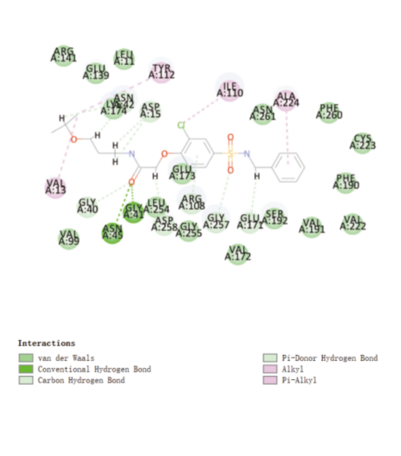

HK-4

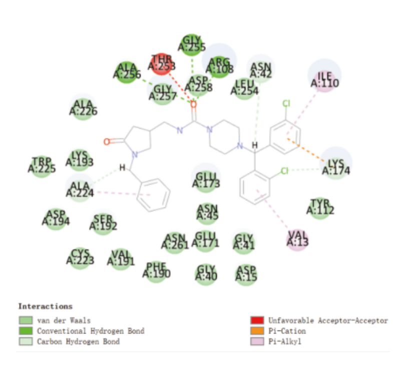

HK-5

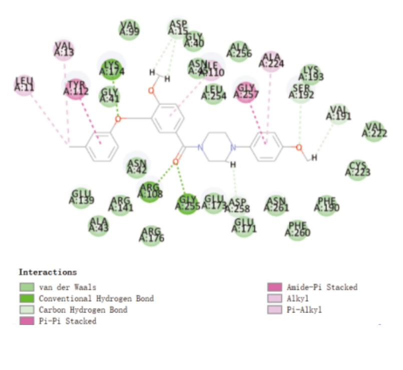

HK-6

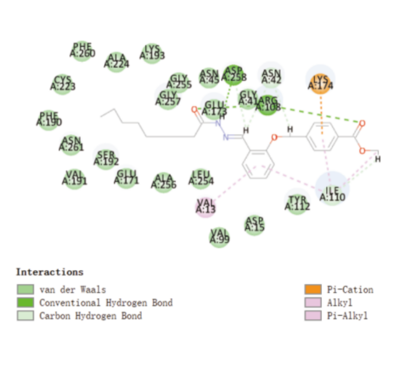

HK-7

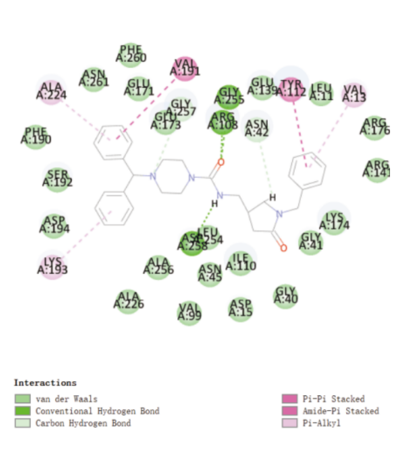

HK-8

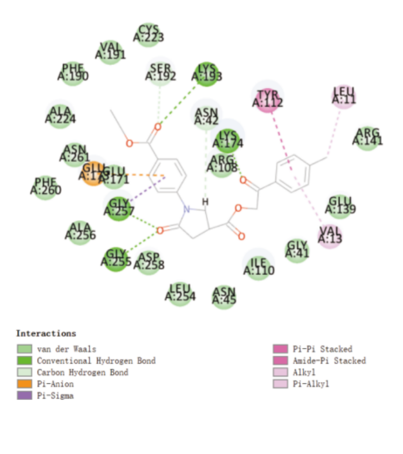

HK-9

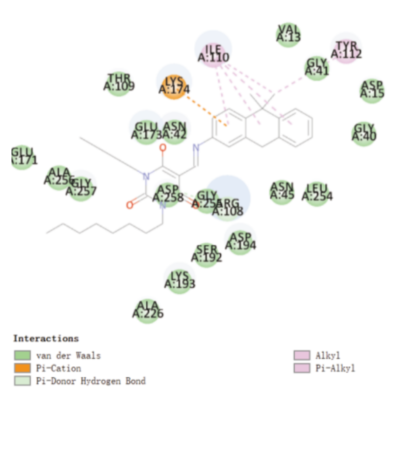

HK-10

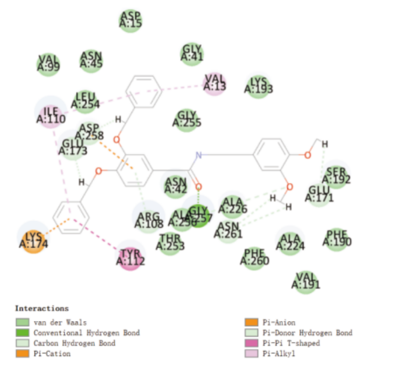

HK-11

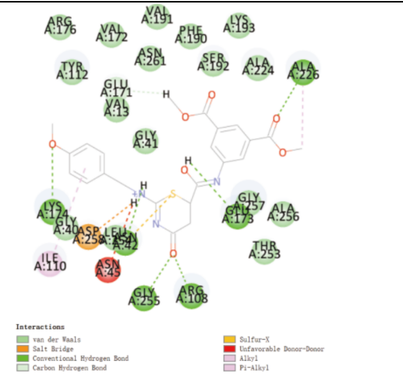

HK-12

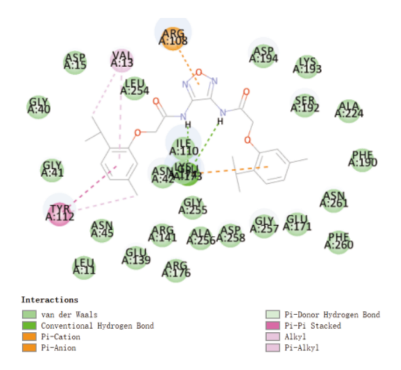

HK-13

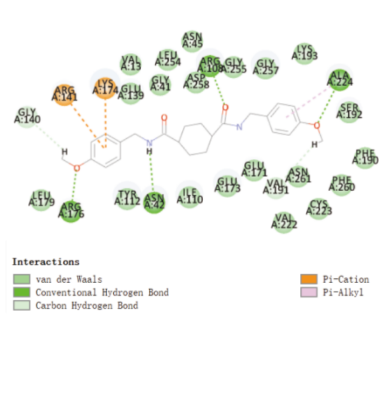

HK-14

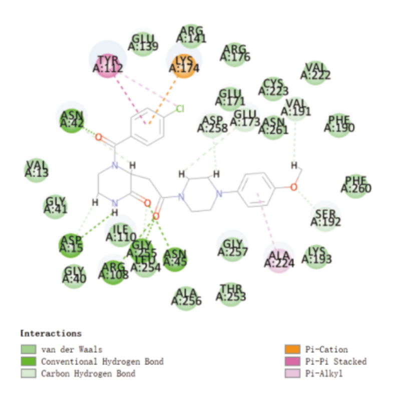

HK-15

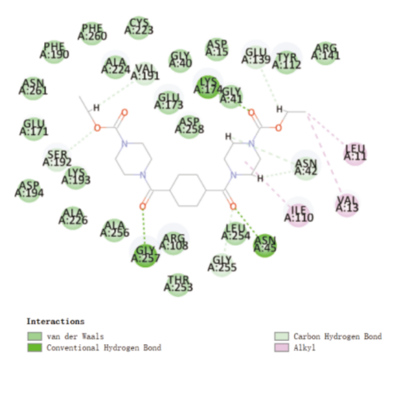

HK-16

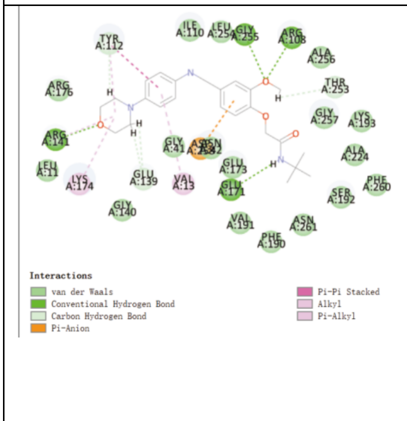

HK-17

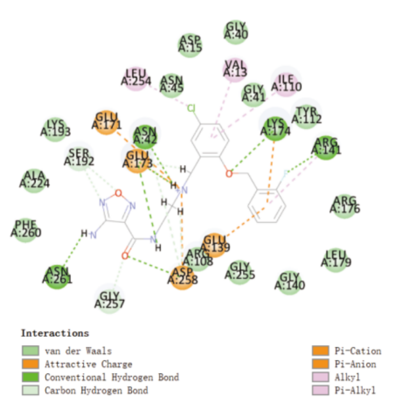

HK-18

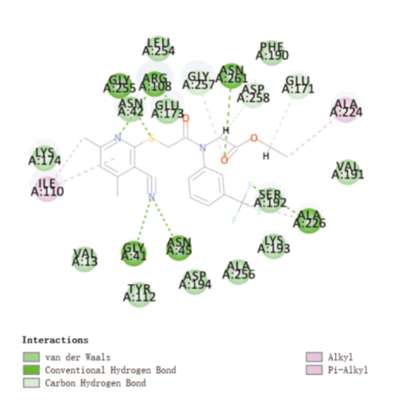

HK-19

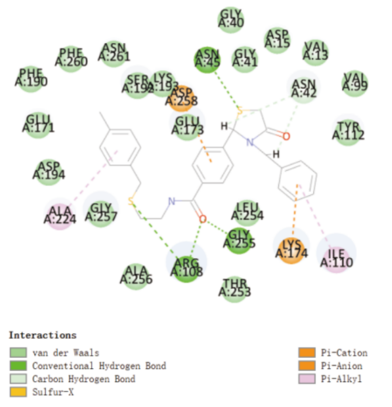

HK-20

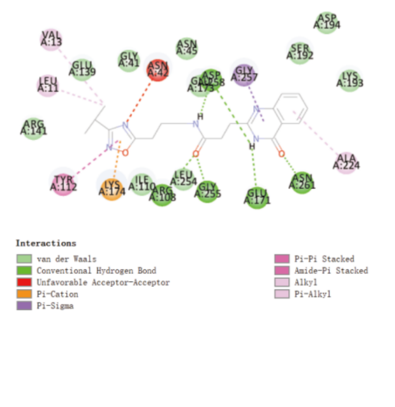

HK-21

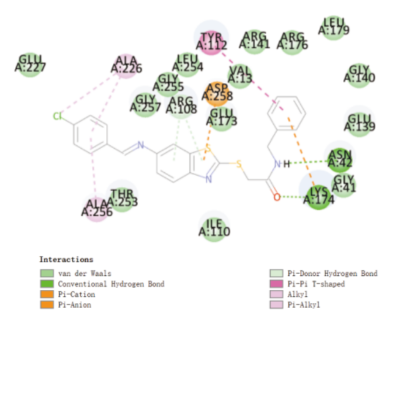

HK-22

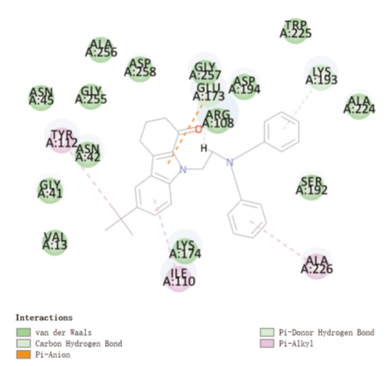

HK-23

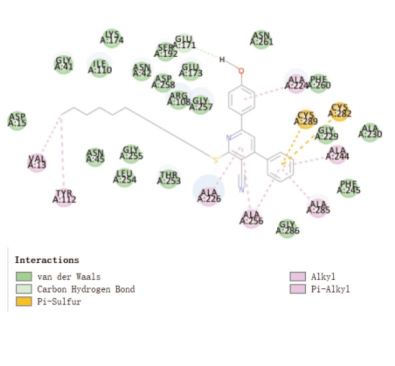

HK-24

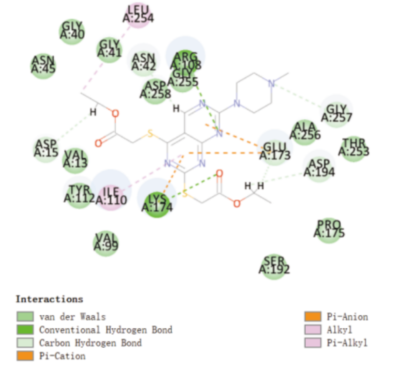

Table S4. Distances of Hydrogen Bonds Formed between HK-1 to HK-24 and Amino Acid Residues at the Protein Active Site.

HK-1	HK-2	HK-3
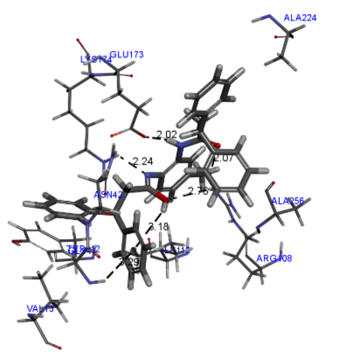	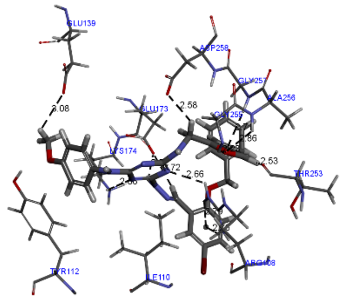	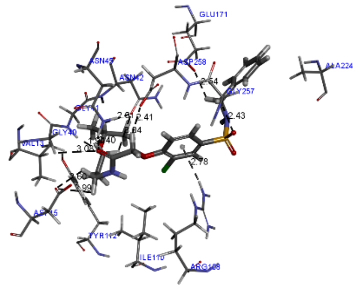
HK-4	HK-5	HK-6
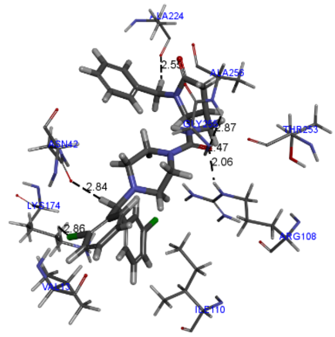	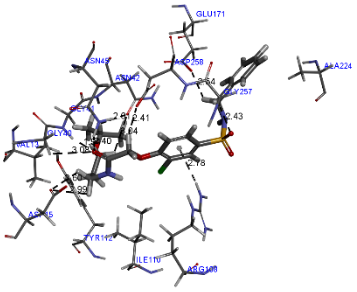	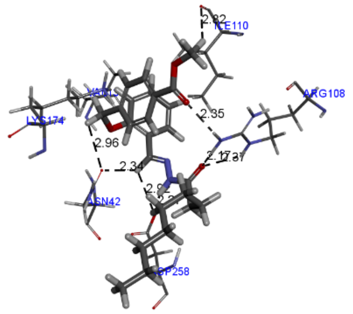
HK-7	HK-8	HK-9
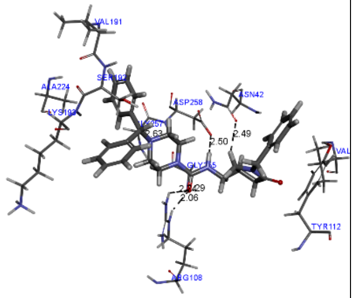	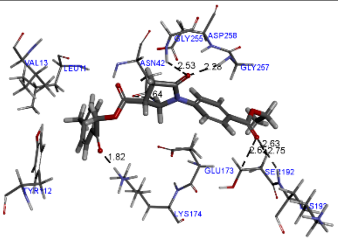	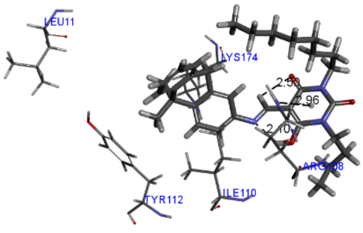
HK-10	HK-11	HK-12
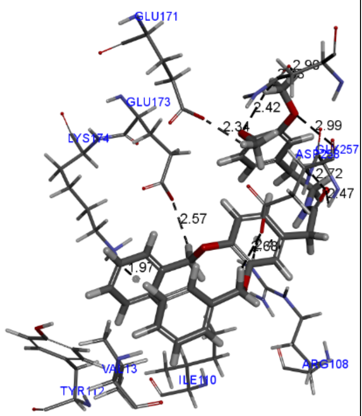	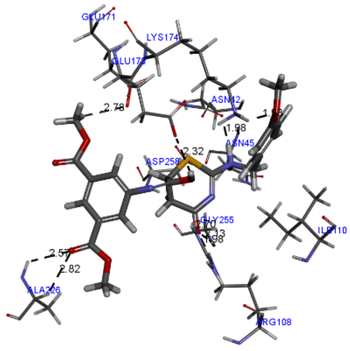	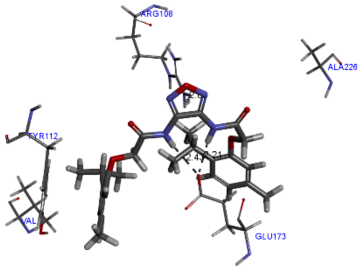

<table>
<tr><td>HK-13</td><td>HK-14</td><td>HK-15</td></tr>
<tr><td>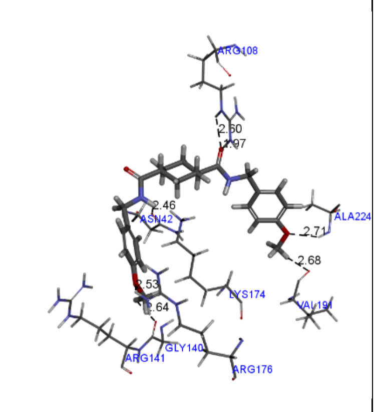
</td><td>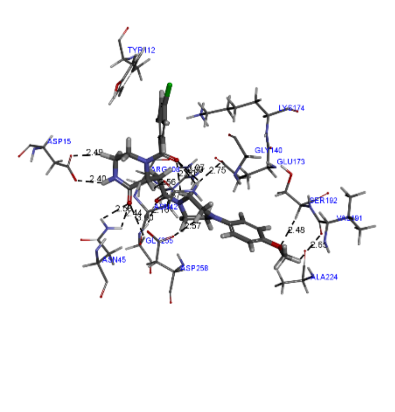</td><td>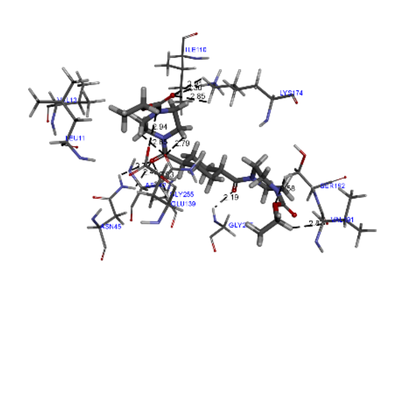</td></tr>
<tr><td>HK-16</td><td>HK-17</td><td>HK-18</td></tr>
<tr><td>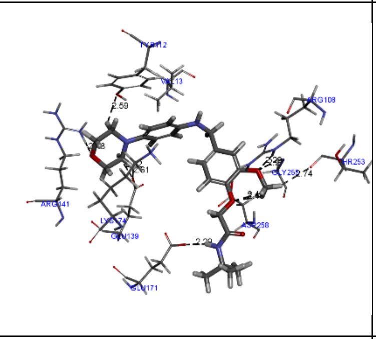</td><td>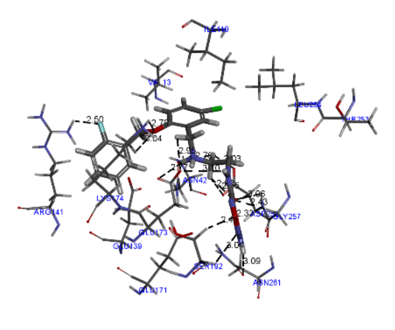</td><td>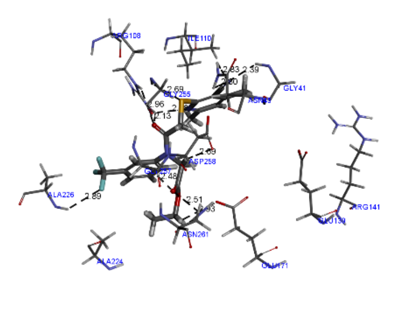</td></tr>
<tr><td>HK-19</td><td>HK-20</td><td>HK-21</td></tr>
<tr><td>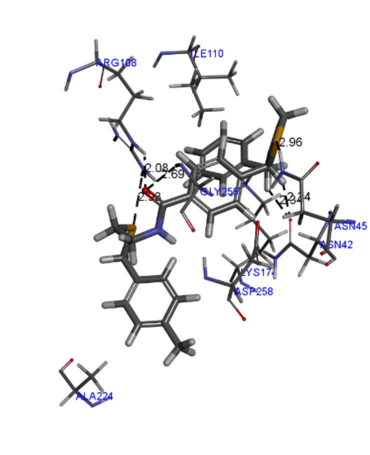
</td><td>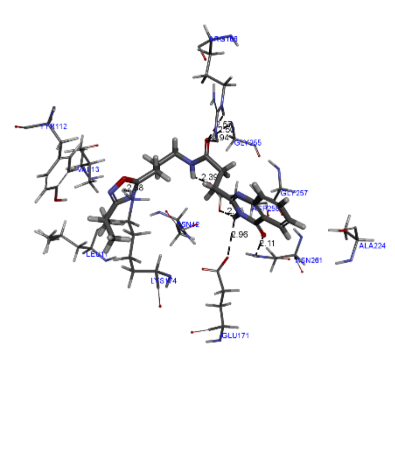</td><td>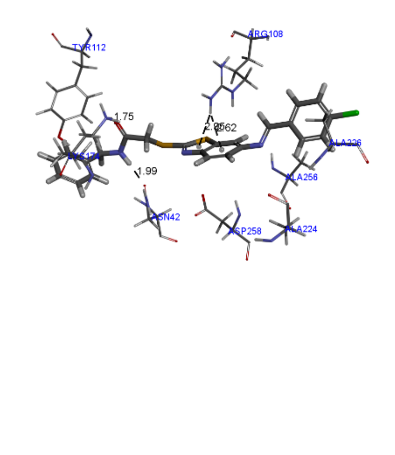
</td></tr>
<tr><td>HK-22</td><td>HK-23</td><td>HK-24</td></tr>
<tr><td>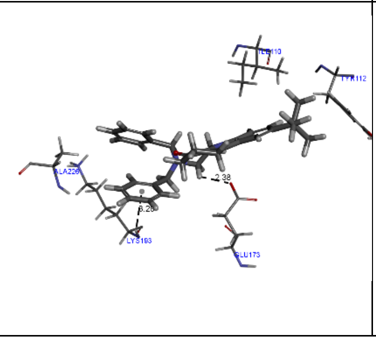</td><td>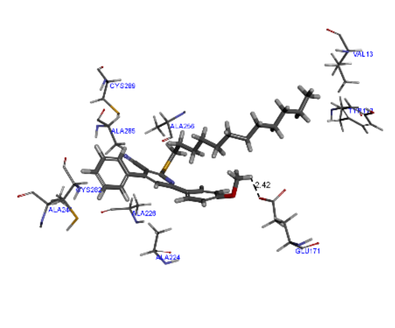</td><td>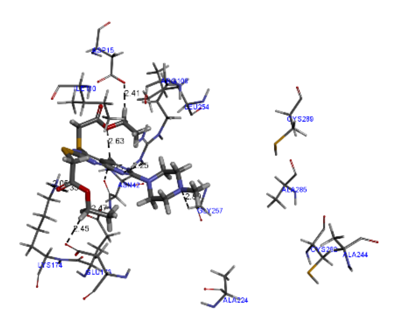</td></tr>
</table>

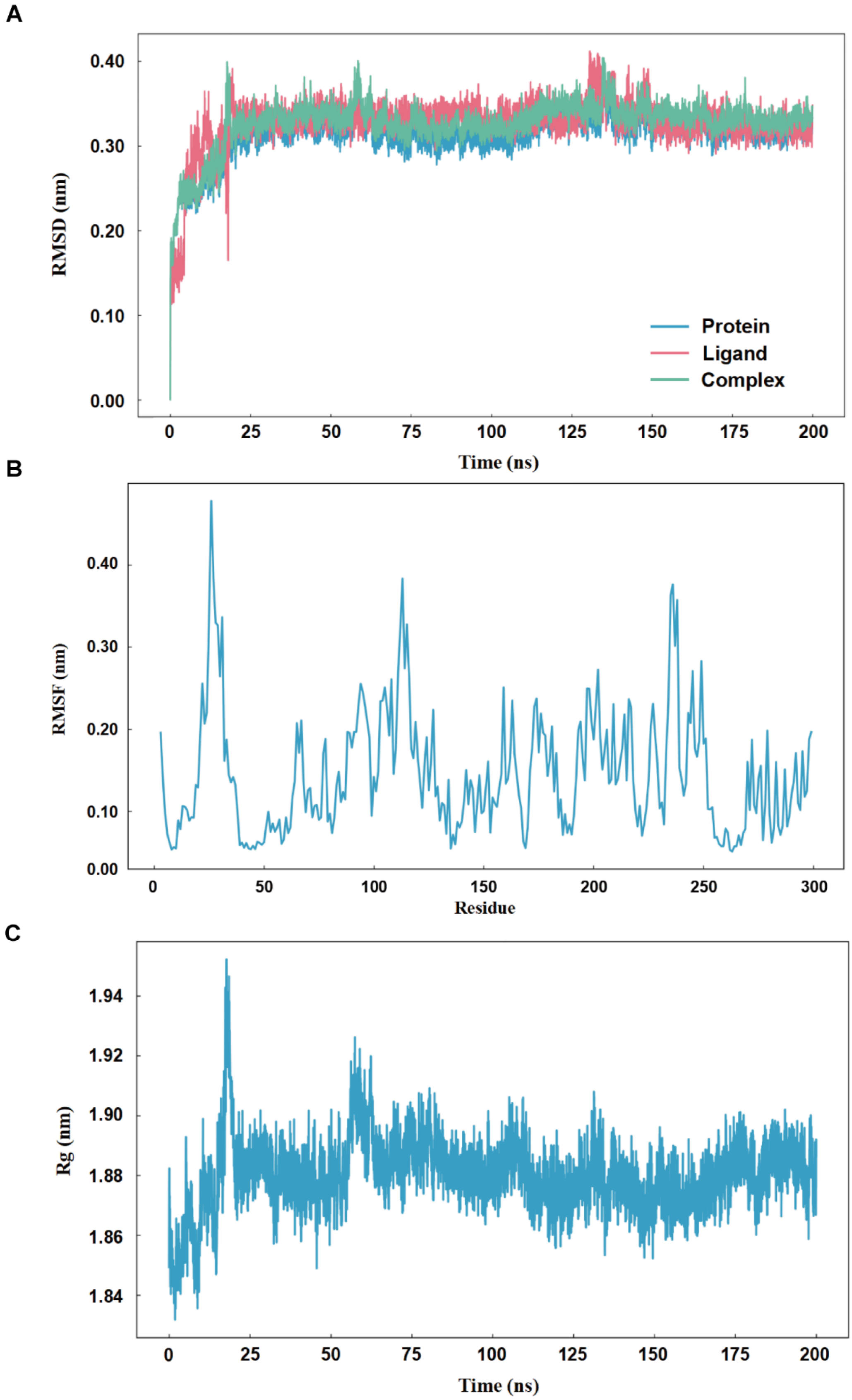

Figure S2. Analysis of Molecular Dynamics of Protein Crystal Complexed with Ligand HK-4. (A) RMSD Changes of the Complex Over Time. (B) RMSF Distribution of Protein Residues. (C) Changes in Rg of Cα Atoms in the Complex.
